# Protective effects of the RAGE inhibitor azeliragon as a potential anti-*Streptococcus pneumoniae* therapeutic in sepsis models

**DOI:** 10.3389/fmed.2026.1793580

**Published:** 2026-04-09

**Authors:** Lei Zhang, Hao Lu, Hao Zhou

**Affiliations:** 1 Department of Pharmacy, Tongxiang First People’s Hospital, Jiaxing, Zhejiang, China; 2 College of Pharmacy, Heze University, Heze, Shandong, China

**Keywords:** antibacterial, azeliragon, *Streptococcus pneumoniae*, sepsis, treatment

## Abstract

The increasing antimicrobial resistance of *Streptococcus pneumoniae* (*S. pneumoniae*) presents a major therapeutic challenge and underscores the need for agents with novel mechanisms of action. Azeliragon is a small-molecule inhibitor of the receptor for advanced glycation end products (RAGE), but its antibacterial activity has not been well defined. In this study, we systematically evaluated the *in vitro* and *in vivo* anti-infective effects of azeliragon against *S. pneumoniae*. Azeliragon exhibited minimum inhibitory concentration (MIC) values predominantly ranging from 4 to 8 μg/mL, with MIC₅₀ and MIC₉₀ values of 4 μg/mL and 8 μg/mL against clinical isolates, respectively. Growth-curve and time-kill assays demonstrated concentration- and time-dependent antibacterial activity, resulting in a marked reduction in viable bacteria within 6–8 h at 4× MIC. Azeliragon also significantly reduced the biomass of mature *S. pneumoniae* biofilms. Mechanistic analyses showed that azeliragon disrupted bacterial membrane integrity and increased intracellular reactive oxygen species levels, indicating that membrane damage and enhanced oxidative stress contribute to its bactericidal effect. *in vivo*, azeliragon conferred significant protection in a mouse infection model of *S. pneumoniae*, improving survival to approximately 60–70%. In a non-lethal infection model, azeliragon markedly reduced bacterial loads in blood and lung tissues and significantly decreased serum levels of pro-inflammatory cytokines, including TNF-α and IL-6. Collectively, these findings provide the first systematic evidence that the RAGE inhibitor azeliragon exerts direct anti-*S. pneumoniae* activity and affords *in vivo* protection, supporting its potential as a novel antibacterial candidate for pneumococcal infections.

## Introduction

*Streptococcus pneumoniae* (*S. pneumoniae*) is a major global cause of invasive bacterial infections. It can induce severe clinical syndromes, including pneumonia, meningitis, and bacteremia/sepsis, with bacteremia- and sepsis-associated mortality being particularly prominent (1, 2). From a global burden perspective, estimates compiled by WHO and the Centers for Disease Control and Prevention (CDC) indicate that *S. pneumoniae* still causes approximately 300,000 deaths annually among children under 5 years of age, most of which occur in resource-limited settings (3, 4). Current clinical management of pneumococcal infections relies primarily on antibiotics, including β-lactams, fluoroquinolones, and macrolides (5, 6). However, in the context of sepsis, patient outcomes depend not only on pathogen clearance but also on host immune dysregulation and progressive organ injury (7, 8). As a result, reliance on conventional antibiotics alone may be insufficient in severe cases, with persistently high mortality rates. In parallel, prevention and treatment of invasive pneumococcal disease are closely linked to vaccination strategies (9). The existence of more than 90 serotypes, with only a subset accounting for most disease burden, underscores the need for continuous surveillance of circulating strains and associated clinical outcomes across regions (10). Improving clinical outcomes in pneumococcal infections therefore remains a critical unmet challenge.

With the long-term and widespread use of antibiotics, antimicrobial resistance in *S. pneumoniae* has become increasingly prominent and represents a major global public health concern (11–13). Surveillance studies have reported rising resistance to penicillins, cephalosporins, and macrolides in many regions, with some lineages exhibiting multidrug-resistant phenotypes (14, 15). Among 3,626 pneumococcal isolates collected in the United States during 2018–2019, macrolide resistance reached 39.5%, and was significantly higher in respiratory isolates than in blood isolates (16). Pneumococcal resistance arises through multiple mechanisms. Alterations in penicillin-binding proteins reduce β-lactam efficacy, while efflux systems and ribosomal target modifications impair macrolide activity (17, 18). In addition, the high genetic plasticity and serotype diversity of *S. pneumoniae* facilitate the acquisition and dissemination of resistance determinants under antibiotic pressure (19, 20). Consequently, single-target agents are more easily overcome by resistance evolution. Reflecting this concern, WHO has included penicillin-non-susceptible *S. pneumoniae* on its priority list for new antibiotic development (21). The spread of resistant strains not only increases the risk of treatment failure and disease recurrence but also imposes substantial healthcare burdens. Antimicrobial resistance therefore represents a central barrier to effective pneumococcal therapy and highlights the urgent need for alternative treatment strategies with novel mechanisms or multi-target activity.

The discovery and development of novel antibacterial agents represent a critical strategy to address the growing crisis of antimicrobial resistance. In recent years, alternative approaches to conventional antibiotics have been explored, including targeting bacterial virulence factors, modulating host immune responses, and disrupting key bacterial metabolic pathways (22, 23). Among these strategies, drug repurposing, also referred to as drug repositioning, has emerged as an efficient and practically feasible model for antibacterial drug development. This approach focuses on re-evaluating small-molecule compounds with established pharmacological and safety profiles for new therapeutic indications (24, 25). Compared with *de novo* compound discovery, repurposing studies benefit from well-characterized pharmacokinetics and manageable safety risks, which can reduce developmental uncertainty and accelerate translational progress (26). The receptor for advanced glycation end products (RAGE) is a membrane-associated pattern recognition receptor that plays a central role in inflammatory signal amplification and immune regulation (27). Aberrant RAGE activation has been implicated in the pathogenesis of multiple diseases, including cancer, inflammatory disorders, and Alzheimer’s disease (28, 29). Azeliragon (TTP488) is a small-molecule RAGE inhibitor with favorable oral bioavailability. It blocks the interaction between RAGE and amyloid-β, attenuates neuroinflammatory responses, and is capable of crossing the blood–brain barrier (30, 31). Azeliragon has been investigated in multiple clinical indications. For Alzheimer’s disease, azeliragon has advanced to phase III clinical trials (30–32). For glioblastoma multiforme, diabetic nephropathy, and COVID-19, it has reached phase II clinical trials (33, 34). Notably, no studies have yet examined the role of azeliragon in antibacterial therapy against *S. pneumoniae*. Given the pivotal role of RAGE in inflammation amplification and tissue injury, this study explored the anti-infective potential of azeliragon in pneumococcal sepsis. We systematically assess its *in vitro* and *in vivo* antibacterial activity and underlying mechanisms, with the aim of providing new insights into therapeutic strategies for invasive pneumococcal infections.

## Materials and methods

### Bacterial strains and reagents

*Streptococcus pneumoniae* American Type Culture Collection (ATCC) 49,619 and the clinical isolates 1057 and 1044 were used for *in vitro* antimicrobial assays, mechanistic studies, and *in vivo* infection models. *Staphylococcus aureus* ATCC 29213, *Staphylococcus epidermidis* ATCC 12228, *Escherichia coli* ATCC 25922, and *Salmonella enterica* ATCC 14028 were used to evaluate the antibacterial spectrum. Clinical isolates were recovered from the laboratory strain repository and cultured on TSA or MHA plates supplemented with 10% fetal bovine serum (FBS). Liquid cultures were grown in tryptic soy broth (TSB) or Mueller–Hinton broth (MHB) containing 10% FBS at 37 °C to the logarithmic phase. Bacterial suspensions were adjusted by centrifugation and resuspension before experiments.

Azeliragon stock solutions (e.g., 10–50 mg/mL) were prepared in an appropriate solvent (dimethyl sulfoxide, 100% DMSO), sterilized through 0.22 μm organic microporous filters, aliquoted, and stored at −20 °C in the dark. Working solutions were prepared by dilution in the corresponding medium, with the final solvent concentration kept constant across all groups (typically ≤1%). An equivalent volume of DMSO was included as the vehicle control in all experiments. Penicillin, tetracycline, erythromycin, ampicillin, cefaclorand, vancomycin were prepared and stored according to the manufacturers’ instructions. Propidium iodide (PI) (C2030S) and reactive oxygen species (ROS) probes (S0034M) were obtained from Shanghai Beyotime Biotechnology Co., Ltd. (Shanghai, China) and prepared according to the kit protocols.

### Determination of minimum inhibitory concentrations

MICs of azeliragon and comparator antibiotics were determined using the standard broth microdilution method (35). Drugs were twofold serially diluted in MHB supplemented with 10% FBS in sterile 96-well plates. Log-phase bacteria were inoculated to a final concentration of 5 × 10^5^ CFU/mL. Sterility and growth controls were included. Plates were incubated at 37 °C for 18 h. Bacterial growth was assessed by visual inspection and OD₆₀₀ measurement. The MIC was defined as the lowest concentration that completely inhibited visible growth. For clinical *S. pneumoniae* isolates, MIC values were ordered to calculate MIC₅₀ and MIC₉₀.

### Dose–response assay and growth curve analysis

*Streptococcus pneumoniae* ATCC 49619 and isolates 1057 and 1044 were adjusted to the same initial density and inoculated into MHB containing 10% FBS and different concentrations of azeliragon (final inoculum: 5 × 10^5^ CFU/mL). For dose–response assays, bacteria were incubated with 0–128 μg/mL azeliragon for 18 h, and OD₆₀₀ was measured. For growth curves, bacteria were cultured with 1, 2, or 4 μg/mL azeliragon or without drug. OD₆₀₀ was recorded at defined time points to assess growth kinetics (36).

### Time-kill assay

Time-kill kinetics were evaluated using log-phase *S. pneumoniae* adjusted to 1 × 10^6^ CFU/mL. Bacteria were mixed 1:1 with azeliragon (4× MIC) or vancomycin (4× MIC). A drug-free control was included. Cultures were incubated at 37 °C. At selected time points, samples were serially diluted and plated on TSA supplemented with 10% FBS. After 18 h incubation, CFU/mL were enumerated and plotted over time (37).

### Biofilm quantification assay

Biofilm biomass was quantified by crystal violet staining. Bacterial suspensions were inoculated into 96-well plates and incubated statically in TSB containing 10% FBS for 24 h to form mature biofilms. After removal of planktonic cells and gentle washing, fresh medium containing azeliragon (4× MIC) or vancomycin (4× MIC) was added for 24 h. Controls received medium alone. Biofilms were stained with crystal violet, washed, and the bound dye was solubilized. Biofilm biomass was quantified by measuring OD₅₇₀ (38).

### Assessment of membrane permeability by PI staining

Log-phase bacteria were adjusted to OD₆₀₀ = 0.5 and treated 1:1 with azeliragon (4× MIC) or vancomycin (4× MIC). A drug-free control was included. After 2 h incubation, cells were collected and stained with PI under dark conditions. Fluorescence intensity was measured at an excitation wavelength of 535 nm and an emission wavelength of 617 nm. Increased PI fluorescence indicated increased membrane permeability (39).

### Measurement of intracellular reactive oxygen species

Intracellular ROS levels were measured using a fluorescent probe. Log-phase bacteria were adjusted to OD₆₀₀ = 0.5 and treated with azeliragon (4× MIC) or vancomycin (4× MIC) for 2 h. Cells were then loaded with the ROS probe, incubated in the dark, washed, and fluorescence intensity was measured using excitation and emission wavelengths of 488 nm and 525 nm, respectively (40).

### Mouse lung infection model

In the mouse lung infection model, mice were anesthetized with isoflurane using an inhalation anesthesia system. The clinical isolate *S. pneumoniae* 1044 was first cultured to the logarithmic phase, collected by centrifugation, and resuspended in sterile saline. Mice were then intranasally inoculated with 50 μL of bacterial suspension at different concentrations (10^6^, 10^7^, or 10^8^ CFU) to establish the lung infection model. Mice in the uninfected control group received the same volume of sterile saline. Survival was monitored for 7 days to determine the appropriate infectious dose for subsequent treatment experiments.

After determining the infectious dose, mice were intranasally challenged with 50 μL of *S. pneumoniae* 1044 suspension at a final concentration of 10^8^ CFU. Twelve hours after infection, mice were randomly divided into four groups (*n* = 10 per group). Mice then received a single intraperitoneal injection of azeliragon (5 or 10 mg/kg) or vancomycin (10 mg/kg). The drug dosage was selected according to previously published study with minor modifications (41) Mice in the bacterial control group received the vehicle, while mice in the uninfected control group received sterile saline. Survival was monitored for 7 days, and Kaplan–Meier survival curves were generated to evaluate the therapeutic efficacy of each treatment.

### Mouse sepsis models and *in vivo* efficacy evaluation

The experimental procedures for the animal infection model were based on previous literature with minor modifications (36, 38). Specific-pathogen-free C57BL/6 mice (6–8 weeks old, 18–22 g; equal numbers of males and females) were obtained from an accredited supplier. Mice were acclimated for 7 days before experiments. *S. pneumoniae* ATCC 49619 and clinical isolate 1044 were grown to log phase, washed, and resuspended in sterile PBS for infection.

For lethal sepsis models, mice were infected intraperitoneally with 1.0 × 10^8^ CFU in 200 μL. Two hours after infection, mice were randomized into groups (*n* = 10 per group) and treated once by intraperitoneal injection with azeliragon (5 or 10 mg/kg) or vancomycin (10 mg/kg). The drug dosage was selected according to previously published study with minor modifications (42). Bacterial control mice received vehicle, and uninfected controls received PBS. Survival was monitored for 7 days, and Kaplan–Meier survival curves were generated.

For non-lethal infection models, mice were infected intraperitoneally with 1.0 × 10^7^ CFU in 200 μL. Two hours later, mice were randomized (*n* = 5 per group) and treated with azeliragon (10 mg/kg) or vancomycin. On day 3 post-infection, mice were euthanized. Blood and lung tissues were collected. Serum was isolated for ELISA measurement of IL-6 and TNF-α. Lungs were weighed, homogenized, serially diluted, and plated on TSA containing 10% FBS. Plates were incubated at 37 °C overnight for CFU enumeration. All animal experiments were approved by Heze University animal ethics committee and conducted in accordance with national animal welfare regulations and ARRIVE guidelines. Animal ethics approval number was 20251229.

### Statistical analysis

All experiments included at least three biological replicates. Data are presented as mean ± SD. Multiple-group comparisons were performed using one-way ANOVA followed by Tukey’s multiple-comparison test. Survival data were analyzed using the Kaplan–Meier method followed by log-rank (Mantel–Cox) test. Statistical analyses were conducted using GraphPad Prism 9.0. A value of *p* < 0.05 was considered statistically significant.

## Results

### Azeliragon exhibits antibacterial activity against *Streptococcus pneumoniae*

**Figure 1:**
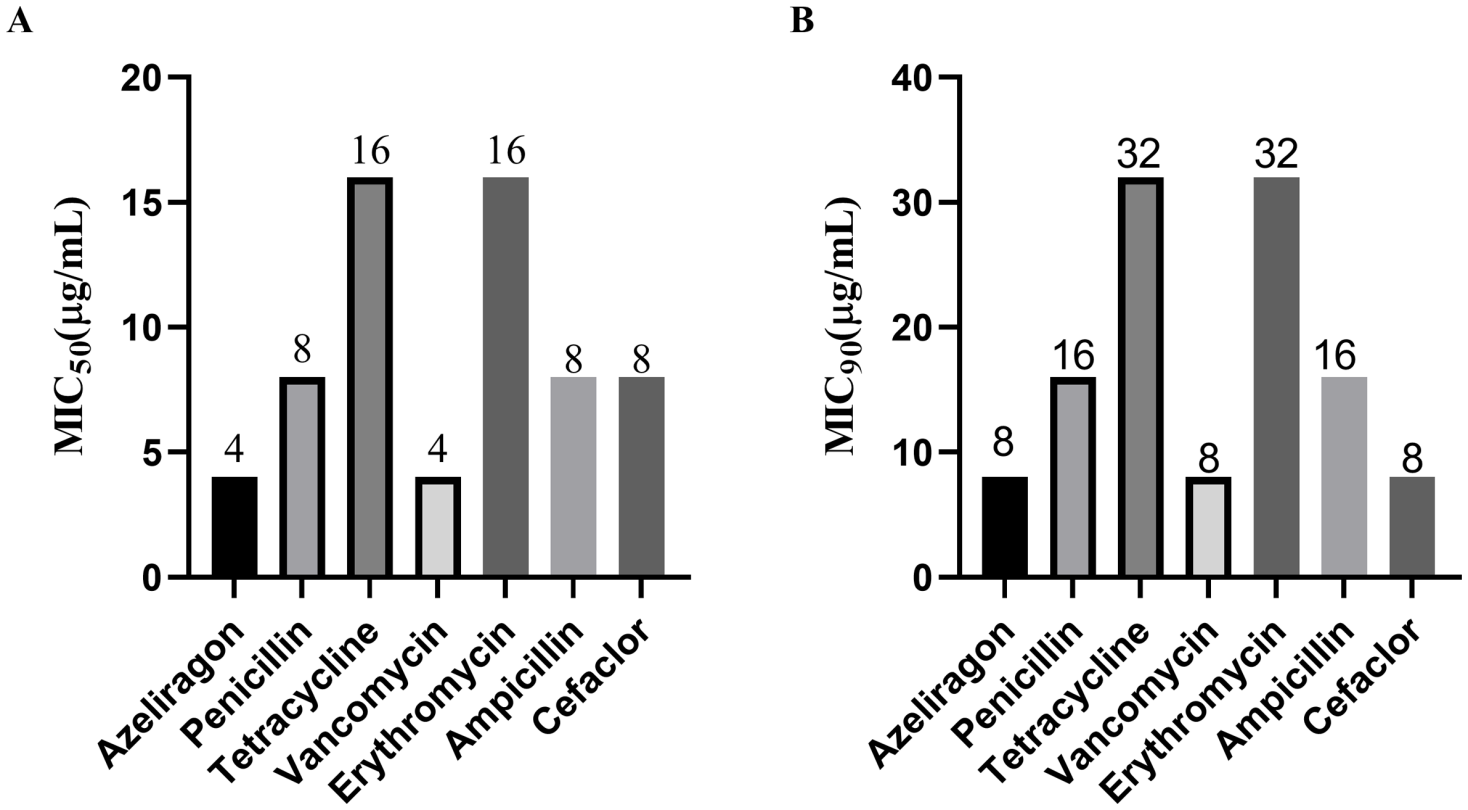
Minimum inhibitory concentrations (MICs) of azeliragon, penicillin, tetracycline, and vancomycin against clinical *S. pneumoniae* isolates were determined using the broth microdilution method. MIC_50_ [the lowest concentration inhibiting 50% of isolates; **(A)**] and MIC_90_ [the lowest concentration inhibiting 90% of isolates; **(B)**] were calculated. Values shown above the bars indicate the corresponding MIC_50_ or MIC_90_ (μg/mL). Data were presented as mean ± SD. The experiments were performed with three independent biological replicates (*n* = 3).

To assess the *in vitro* antibacterial activity of azeliragon, minimum inhibitory concentrations (MICs) were determined by broth microdilution against representative Gram-positive and Gram-negative bacteria and clinical isolates of *S. pneumoniae*. Azeliragon exhibited stable antibacterial activity against several Gram-positive bacteria. For the reference strains, the MIC of azeliragon against *S. aureus* ATCC 29213, *S. epidermidis* ATCC 12228, and *S. pneumoniae* ATCC 49619 was 4 μg/mL, indicating good inhibitory activity against Gram-positive pathogens (Table 1). In contrast, azeliragon showed weak activity against Gram-negative bacteria, with MIC values >128 μg/mL against *E. coli* ATCC 25922 and *S. enterica* ATCC 14028 (Table 1). Among 16 clinical isolates of *S. pneumoniae*, the MIC values of azeliragon were narrowly distributed between 4 and 8 μg/mL, indicating stable antibacterial activity across isolates (Table 1). In comparison, commonly used antibiotics showed broader MIC ranges. Penicillin, tetracycline, and erythromycin ranged from 8 to 64 μg/mL, while ampicillin and cefaclor ranged from 2–32 μg/mL and 1–16 μg/mL, respectively (Table 1), suggesting strain-to-strain variability and potential resistance. In contrast, vancomycin remained highly active, with MIC values of 2–8 μg/mL (Table 1). Based on the MIC distribution analysis, MIC₅₀ and MIC₉₀ values were calculated to compare overall antibacterial potency. For azeliragon, the MIC₅₀ was 4 μg/mL and the MIC₉₀ was 8 μg/mL, indicating effective inhibition of at least 50 and 90% of clinical isolates within this concentration range (Figures 1A,B). In contrast, penicillin and ampicillin showed MIC₅₀ and MIC₉₀ values of 8 μg/mL and 16 μg/mL, respectively. Tetracycline and erythromycin exhibited further reduced activity, with MIC₅₀ and MIC₉₀ values of 16 μg/mL and 32 μg/mL (Figures 1A,B). Cefaclor showed MIC₅₀ and MIC₉₀ values of 8 μg/mL and 8 μg/mL, respectively (Figures 1A,B). Vancomycin showed MIC₅₀ and MIC₉₀ values of 4 μg/mL and 8 μg/mL, which were comparable to those of azeliragon (Figures 1A,B). Overall, these results demonstrated that azeliragon possessed measurable *in vitro* antibacterial activity against *S. pneumoniae*, with an antibacterial spectrum biased toward Gram-positive bacteria.

**Table 1:** MICs of drugs for strain used in the study.

Strains	Source	MIC (μg/mL)						
		Azeliragon	Penicillin	Tetracycline	Vancomycin	Erythromycin	Ampicillin	Cefaclor
*S. aureus* ATCC 29213		4	0.5	1	0.5	0.5	1	2
*S. epidermidis* ATCC 12228		4	0.5	1	1	1	0.5	1
*S. pneumoniae* ATCC 49619		4	0.5	0.5	0.25	0.125	0.25	1
*E. coli* ATCC25922		>128	—	2	—	—	2	2
*S. enterica* ATCC14028		>128	—	1	—	—	2	2
*S. pneumoniae* 1041	China (Jiaxing)	8	8	8	4	16	8	4
*S. pneumoniae* 1057	China (Jiaxing)	4	16	32	4	32	16	8
*S. pneumoniae* 1050	China (Jiaxing)	4	8	16	2	8	8	4
*S. pneumoniae* 1153	China (Jiaxing)	4	4	8	8	8	4	8
*S. pneumoniae* 1042	China (Jiaxing)	8	2	16	4	16	2	1
*S. pneumoniae* 1024	China (Jiaxing)	8	8	16	8	32	16	8
*S. pneumoniae* 1031	China (Jiaxing)	8	16	32	8	32	16	8
*S. pneumoniae* 1044	China (Jiaxing)	8	16	64	8	64	32	16
*S. pneumoniae* 1053	China (Jiaxing)	4	16	16	8	32	8	4
*S. pneumoniae* 1021	China (Jiaxing)	8	8	16	8	32	16	8
*S. pneumoniae* 1032	China (Jiaxing)	4	8	16	2	16	8	4
*S. pneumoniae* 1034	China (Jiaxing)	8	16	32	8	16	8	8
*S. pneumoniae* 1026	China (Jiaxing)	8	8	16	2	16	8	4
*S. pneumoniae* 1028	China (Jiaxing)	4	8	8	2	8	16	8
*S. pneumoniae* 1406	China (Jiaxing)	4	16	16	8	32	16	8
*S. pneumoniae* 1141	China (Jiaxing)	4	16	16	4	16	16	8

### Azeliragon inhibits the *in vitro* growth of *Streptococcus pneumoniae* in a concentration- and time-dependent manner

**Figure 2:**
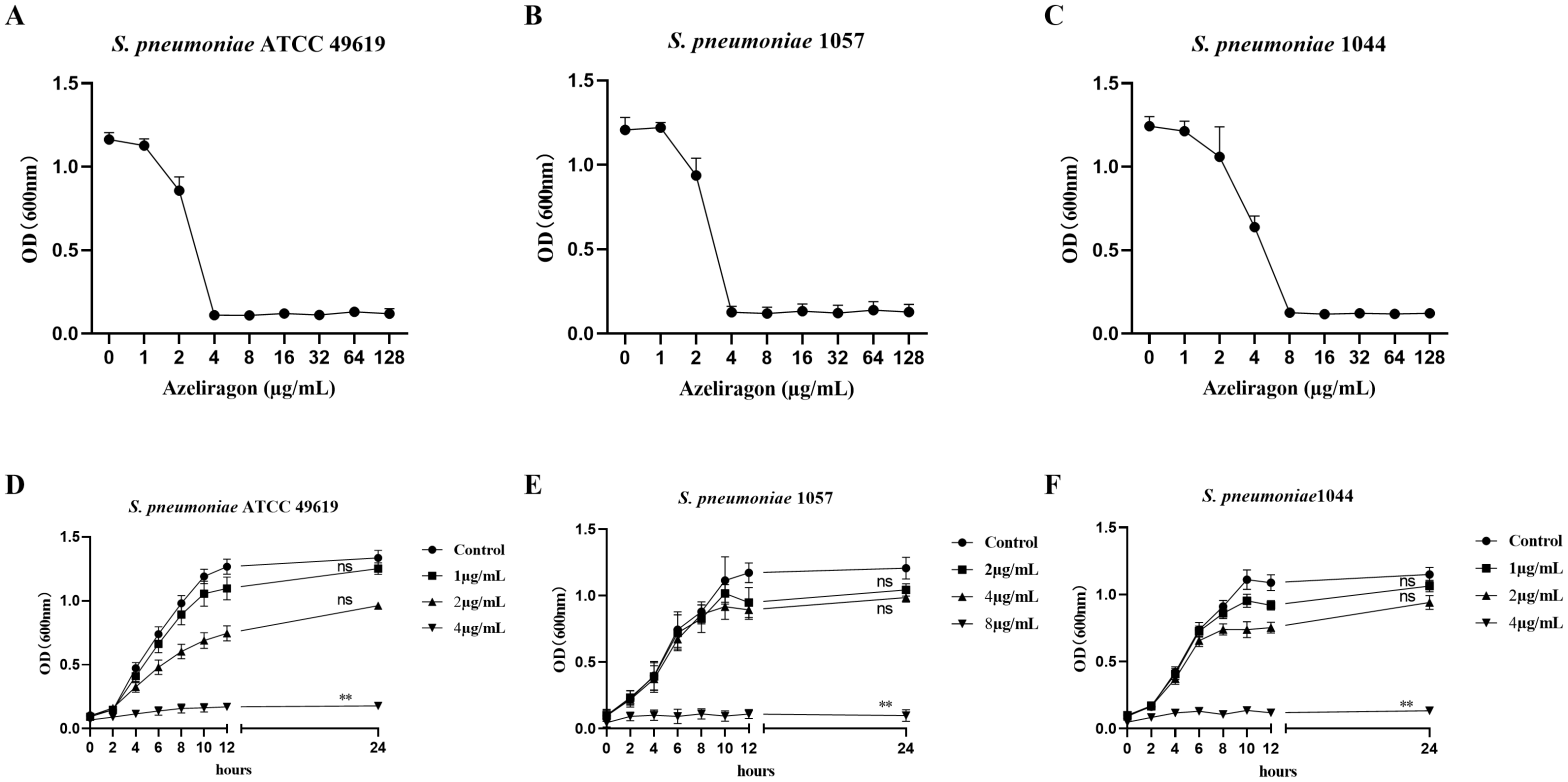
Effects of azeliragon on the *in vitro* growth of *S. pneumoniae*. Dose–response relationships of *S. pneumoniae* ATCC 49619 **(A)**, *S. pneumoniae* 1057 **(B)**, and *S. pneumoniae* 1044 **(C)** following treatment with increasing concentrations of azeliragon. Bacterial growth was measured after 18 h of incubation and expressed as OD_600_. Effects of azeliragon on the growth kinetics of *S. pneumoniae* ATCC 49619 **(D)**, *S. pneumoniae* 1057 **(E)**, and *S. pneumoniae* 1044 **(F)**. Bacteria were cultured under control conditions or with different concentrations of azeliragon, and OD_600_ was recorded at indicated time points to generate growth curves. Data were presented as mean ± SD. The experiments were performed with three independent biological replicates (*n* = 3). “ns” represented no significant difference, ^**^p < 0.01.

To further assess the effects of azeliragon on pneumococcal growth, the reference strain *S. pneumoniae* ATCC 49619 and two clinical isolates (1057 and 1044) were examined under different drug concentrations. In dose–response experiments, azeliragon showed clear concentration-dependent inhibition against all three strains (Figures 2A–C). At concentrations of 4–8 μg/mL, bacterial growth was markedly suppressed, with OD₆₀₀ values approaching baseline levels (Figures 2A–C). At higher concentrations (≥16 μg/mL), growth of all three strains was almost completely inhibited (Figures 2A–C). The inhibition patterns were consistent among strains, indicating stable concentration-dependent activity. Time-course growth analyses were performed to evaluate the effects of azeliragon on bacterial growth kinetics. In untreated controls, all three strains exhibited typical exponential and stationary growth phases (Figures 2D–F). In contrast, azeliragon treatment caused pronounced growth inhibition in a concentration-dependent manner. At lower concentrations (1–2 μg/mL), bacterial growth was slowed but cells were still able to enter the exponential phase (Figures 2D–F). When the concentration reached 4 μg/mL or higher, growth was strongly suppressed, and OD₆₀₀ values remained low throughout the observation period (Figures 2D–F). Because the MIC values differed among the tested strains, a fixed concentration (e.g., 4 μg/mL) was not applied to all strains to avoid misleading comparisons. This pattern was observed in both the reference strain and the clinical isolates. Overall, these results demonstrated that azeliragon inhibited the *in vitro* growth of *S. pneumoniae* in a concentration- and time-dependent manner across strains from different sources, providing further evidence for its anti-pneumococcal activity.

### Azeliragon exhibits time-dependent bactericidal activity and effectively reduces biofilms of *Streptococcus pneumoniae*

**Figure 3:**
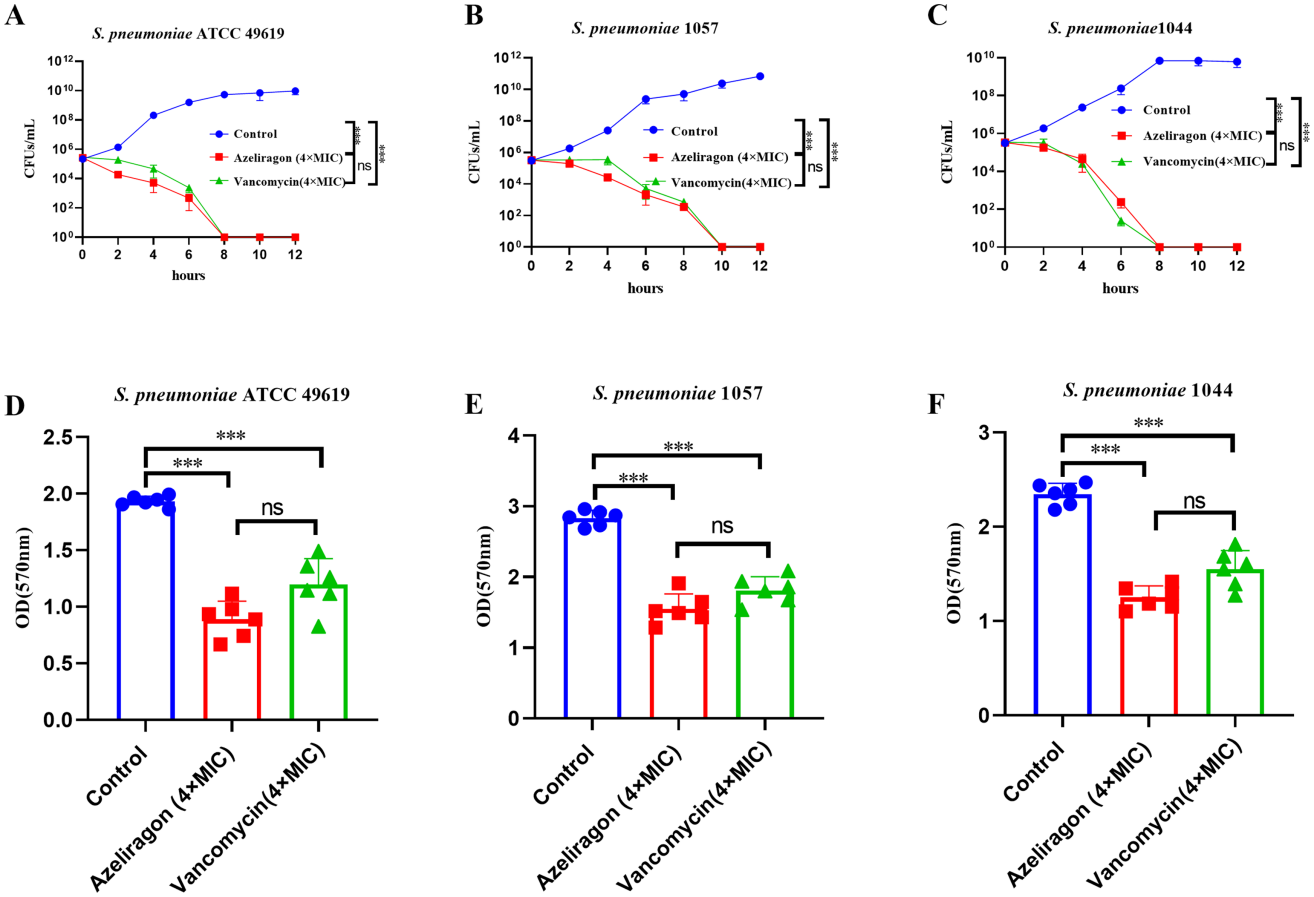
Time-kill activity and biofilm disruption effects of azeliragon against *S. pneumoniae*. Time-kill curves of *S. pneumoniae* ATCC 49619 **(A)**, *S. pneumoniae* 1057 **(B)**, and *S. pneumoniae* 1044 **(C)** treated with azeliragon (4× MIC). Bacteria were cultured under control conditions or treated with azeliragon (4× MIC) or vancomycin (4× MIC), and viable counts (CFU/mL) were determined at indicated time points. Effects of azeliragon (4× MIC) on biofilm biomass of *S. pneumoniae* ATCC 49619 **(D)**, *S. pneumoniae* 1057 **(E)**, and *S. pneumoniae* 1044 **(F)**. Mature biofilms were treated with the indicated drugs for 24 h and quantified by crystal violet staining. Results are expressed as OD₅₇₀. Data were presented as mean ± SD. Data were presented as mean ± SD. The experiments were performed with six independent biological replicates (*n* = 6). Group comparisons for biofilm assays were analyzed by one-way ANOVA followed by Tukey’s multiple-comparison test. “ns” represented no significant difference, ^***^p < 0.001.

To further characterize the bactericidal kinetics of azeliragon and its effects on biofilms, time-kill assays were performed at 4× MIC, and biofilm reduction was evaluated in parallel. Using 4× MIC allows rapid induction of antibacterial effects, reducing potential interference from prolonged bacterial growth. In time-kill experiments, all three *S. pneumoniae* strains in the untreated control group showed continuous growth, with colony-forming units (CFU/mL) increasing over time (Figures 3A–C). In contrast, treatment with azeliragon at 4× MIC led to a progressive decline in viable bacteria, demonstrating a clear time-dependent bactericidal effect (Figures 3A–C). For the ATCC 49619 strain, bacterial counts decreased to the limit of detection within 8 h and remained low throughout the subsequent observation period (until 12 h) (Figure 3A). Similar trends were observed for the clinical isolates 1057 and 1044, in which CFU/mL values decreased markedly over time (Figures 3B,C). The overall killing kinetics were comparable to those observed with vancomycin at 4× MIC. These results indicated that azeliragon exerted sustained and stable time-dependent bactericidal activity against *S. pneumoniae in vitro*. The ability of azeliragon to disrupt pneumococcal biofilms was further assessed (Figures 3D–F). Compared with the control group, azeliragon treatment at 4× MIC significantly disrupted the biomass of mature biofilms formed by all three *S. pneumoniae* strains, as evidenced by markedly lower OD₅₇₀ values following crystal violet staining (Figures 3D–F). This effect was consistently observed in the reference strain and both clinical isolates. The extent of biofilm disruption was comparable to that achieved with vancomycin at 4× MIC (Figures 3D–F). Statistical analysis confirmed significant differences between azeliragon-treated and control groups. Taken together, these findings demonstrated that azeliragon not only exerted time-dependent bactericidal activity against *S. pneumoniae in vitro* but also significantly disrupted pneumococcal biofilm biomass, supporting its potential activity in complex infection settings.

### Azeliragon contributes to bactericidal activity by disrupting membrane integrity and inducing oxidative stress

**Figure 4:**
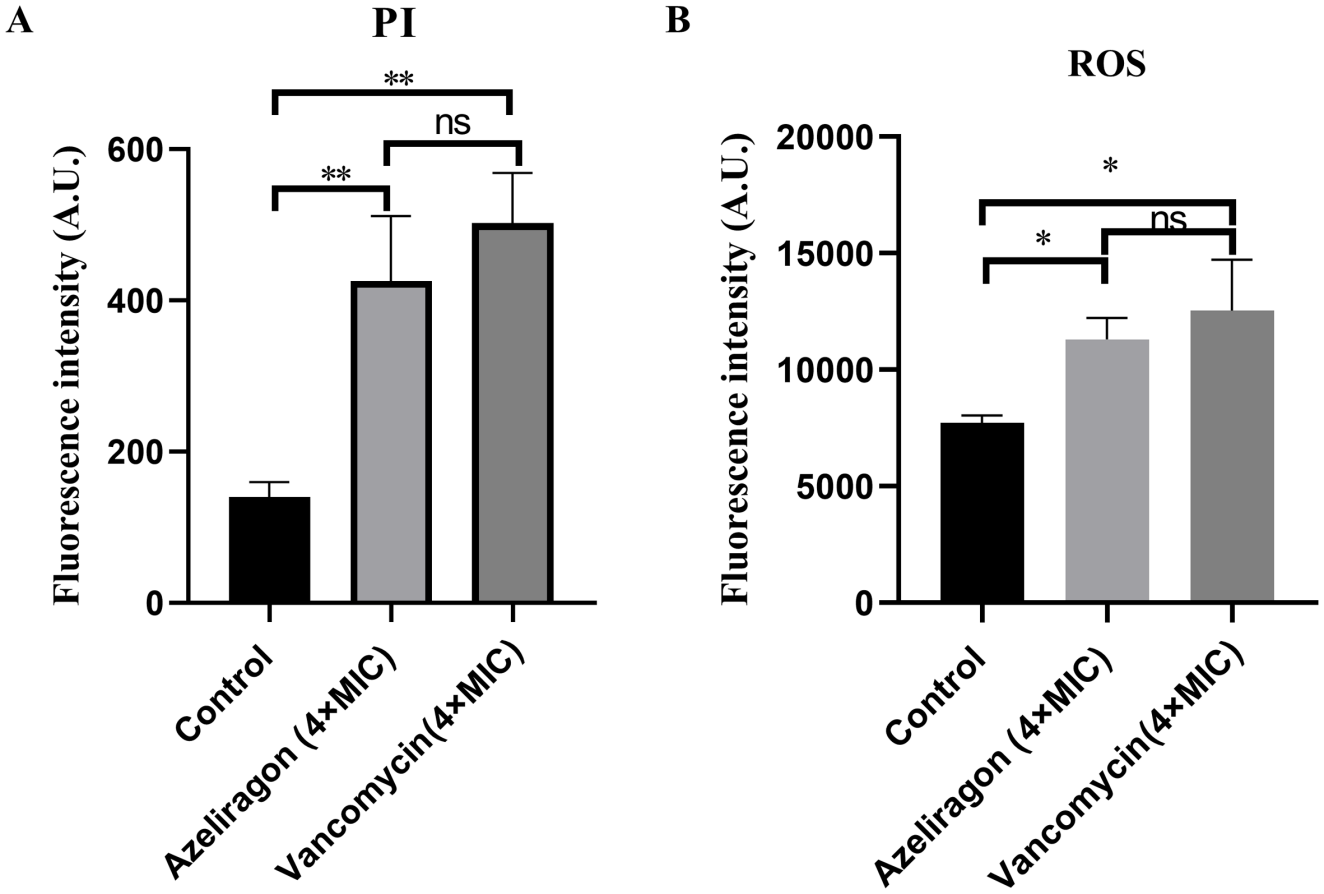
Effects of azeliragon on membrane integrity and oxidative stress in *S. pneumoniae*. **(A)** Propidium iodide (PI) staining was used to assess membrane permeability. Bacteria were treated under control conditions or with azeliragon (4× MIC) or vancomycin (4× MIC). Fluorescence intensity (A.U.) was measured to evaluate membrane integrity. **(B)** Intracellular oxidative stress was assessed using a reactive oxygen species (ROS) fluorescent probe. Bacteria were treated as indicated, and ROS fluorescence intensity (A.U.) was quantified. Data were presented as mean ± SD. Group comparisons were performed using one-way ANOVA followed by Tukey’s multiple-comparison test. Data were presented as mean ± SD. The experiments were performed with three independent biological replicates (*n* = 3). “ns” represented no significant difference, ^*^p < 0.05, ^**^p < 0.01.

To investigate the bactericidal mechanism of azeliragon, bacterial membrane integrity and intracellular oxidative stress were evaluated using PI staining and reactive oxygen species (ROS) fluorescent probes. Compared with the control group, treatment with azeliragon at 4× MIC significantly increased PI fluorescence intensity in *S. pneumoniae*, indicating compromised membrane integrity and increased membrane permeability (Figure 4A). Vancomycin at 4× MIC, used as a positive control, induced a similar increase in PI fluorescence (Figure 4A). These findings suggested that azeliragon disrupted the bacterial membrane barrier, which may contribute to its *in vitro* bactericidal activity. Intracellular ROS levels were further measured to evaluate whether azeliragon induces oxidative stress. As shown in Figure 4B, azeliragon treatment at 4× MIC markedly increased ROS fluorescence intensity compared with the control group, indicating elevated intracellular ROS levels (Figure 4B). Vancomycin at 4× MIC also induced ROS production, with fluorescence intensity comparable to that observed in the azeliragon-treated group (Figure 4B). Excessive ROS accumulation can cause oxidative damage to proteins, lipids, and nucleic acids, thereby exacerbating cellular dysfunction. Taken together, the PI staining and ROS assays indicated that azeliragon not only disrupted membrane integrity but also induced pronounced oxidative stress in *S. pneumoniae*. These effects may act synergistically to mediate its bactericidal activity. These results provided initial experimental evidence for the anti-pneumococcal mechanism of azeliragon and lay the foundation for further mechanistic studies.

### Azeliragon significantly improves survival in mouse lung infection model and mouse model of pneumococcal sepsis

**Figure 5:**
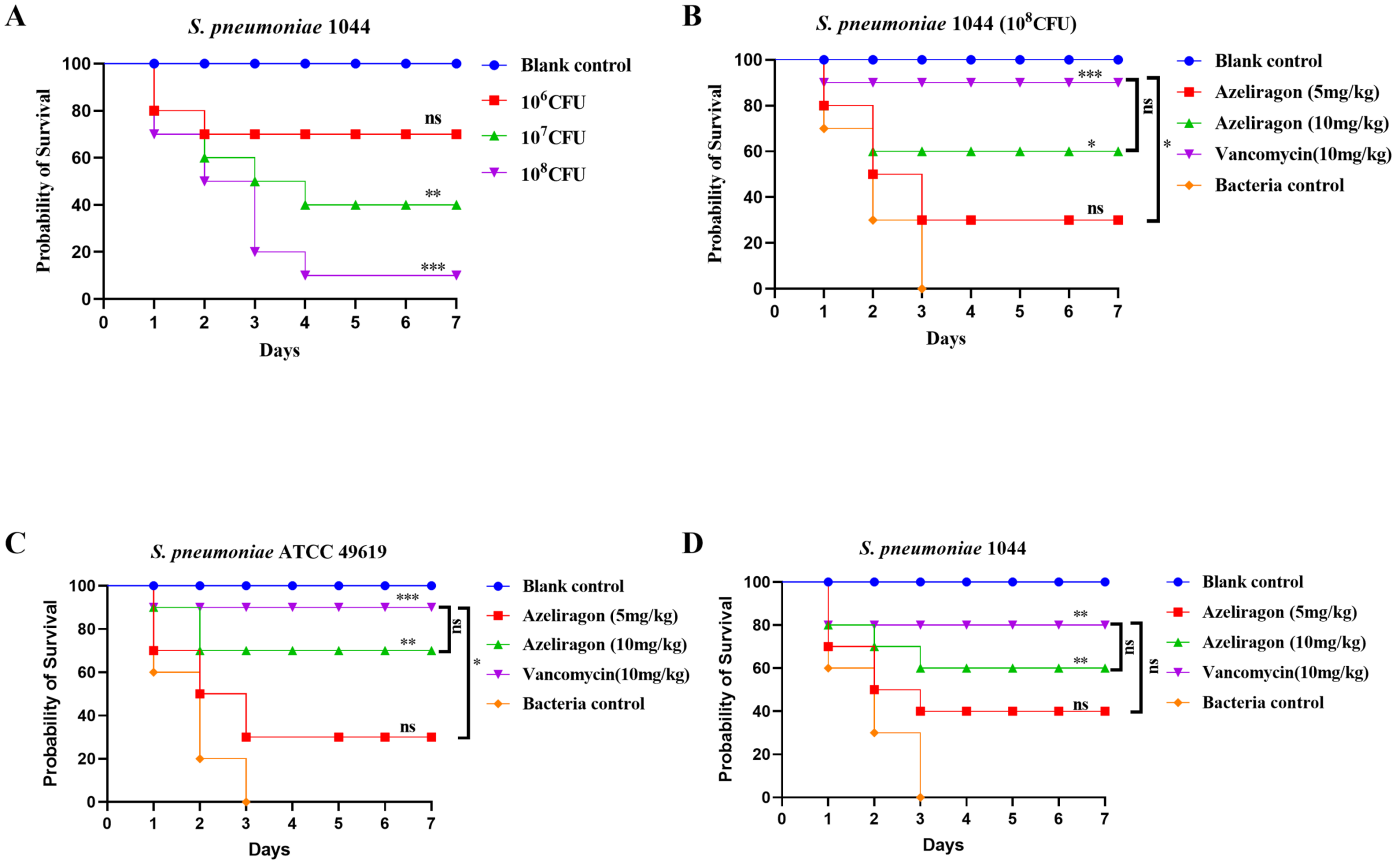
*In vivo* protective effects of azeliragon in a mouse infection model. **(A)** Determination of the infectious dose of *S. pneumoniae* 1044. **(B)** Therapeutic efficacy of azeliragon in the pneumonia model. **(C)** Seven-day survival curves of mice with sepsis induced by *S. pneumoniae* ATCC 49619. Mice received vehicle control, bacterial control, azeliragon (5 mg/kg or 10 mg/kg), or vancomycin (10 mg/kg). Each group contained 10 mice. Survival was monitored continuously. **(D)** Seven-day survival curves of mice with sepsis induced by the clinical *S. pneumoniae* isolate 1044. Experimental groups and treatments were identical to those in **(A)**. Survival data were plotted using the Kaplan–Meier method, and statistical significance was determined using the log-rank (Mantel–Cox) test. “ns” represented no significant difference, ^*^p < 0.05, ^**^p < 0.01, and ^***^p < 0.001.

To establish a mouse pneumonia model, mice were intranasally infected with different doses of *S. pneumoniae* 1044 and monitored for 7 days. As shown in Figure 5A, all mice in the uninfected control group survived throughout the observation period (100%). Infection with 10^6^ CFU resulted in relatively low mortality, with about 70% survival at day 7 (Figure 5A), whereas increasing the dose to 10^7^ CFU reduced survival to approximately 40% (Figure 5A). At 10^8^ CFU, mortality occurred more rapidly and survival decreased to about 10% by day 7 (Figure 5A), indicating a clear dose-dependent relationship. Therefore, 10^8^ CFU was selected for subsequent therapeutic evaluation. As shown in Figure 5B, treatment efficacy was assessed using this infection dose. All mice in the bacterial control group died within 3 days, resulting in a final survival rate of 0% (Figure 5B). In contrast, azeliragon treatment improved survival in a dose-dependent manner, with survival rates of about 30% (5 mg/kg) and 60% (10 mg/kg) at the end of the 7-day observation period (Figure 5B). The positive control vancomycin (10 mg/kg) showed stronger efficacy, with a final survival rate of approximately 90% (Figure 5B). Mice in the uninfected control group maintained 100% survival throughout the experiment (Figure 5B). To futher evaluate the *in vivo* therapeutic efficacy of azeliragon, mouse sepsis models were established using *S. pneumoniae* ATCC 49619 and the clinical isolate 1044. Survival outcomes were compared among mice treated with different doses of azeliragon or vancomycin. In the ATCC 49619 infection model, mice in the bacterial control group died rapidly within 2–3 days after infection, with a 7-day survival rate of 0%, confirming the lethality of the model (Figure 5C). Azeliragon treatment significantly improved survival in a dose-dependent manner (Figure 5C). The 7-day survival rate was approximately 30% in the 5 mg/kg group and increased to about 70% in the 10 mg/kg group (Figure 5C). Vancomycin (10 mg/kg), used as a positive control, provided strong protection, with a 7-day survival rate of approximately 90% (Figure 5C). All mice in the uninfected control group survived throughout the observation period. In the model infected with the clinical isolate 1044, a similar survival pattern was observed. All mice in the bacterial control group died shortly after infection, resulting in a 7-day survival rate of 0% (Figure 5D). Azeliragon treatment again significantly improved survival, with a 7-day survival rate of approximately 40% at 5 mg/kg and about 60% at 10 mg/kg (Figure 5D). Vancomycin treatment yielded a higher protective effect, with a 7-day survival rate of approximately 80% (Figure 5D). No mortality was observed in the uninfected control group. Collectively, these results demonstrated that azeliragon significantly enhanced host survival in mouse models of pneumococcal sepsis. The protective effect was dose dependent and consistent across infections caused by strains from different sources, supporting the potential of azeliragon as a therapeutic candidate for pneumococcal sepsis.

### Azeliragon reduces bacterial burden and attenuates inflammation in a non-lethal pneumococcal infection model

**Figure 6:**
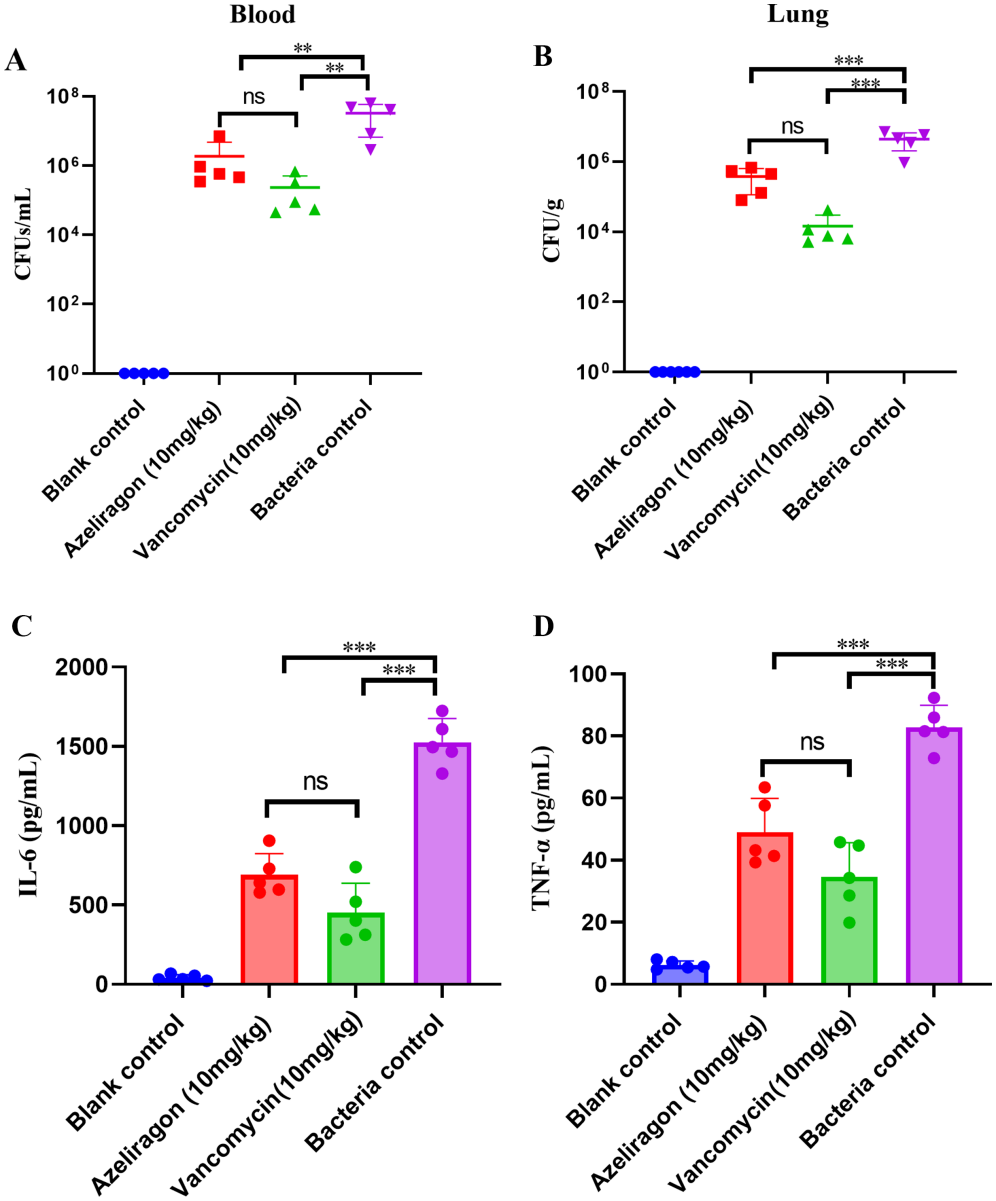
Effects of azeliragon on tissue bacterial burden and inflammatory cytokines in a non-lethal mouse model of *S. pneumoniae* infection. **(A)** Bacterial loads in blood (CFU/mL). **(B)** Bacterial loads in lung tissue (CFU/g). **(C)** Serum interleukin-6 (IL-6) levels (pg/mL). **(D)** Serum tumor necrosis factor-α (TNF-α) levels (pg/mL). Mice received vehicle control, bacterial control, azeliragon (10 mg/kg), or vancomycin (10 mg/kg). Blood and lung samples were collected for quantitative bacterial culture. Serum cytokine levels were measured by ELISA. Data are presented as mean ± SD. Group comparisons were performed using one-way ANOVA followed by Tukey’s multiple-comparison test. “ns” represented no significant difference, ^**^p < 0.01 and ^***^p < 0.001.

To further evaluate the *in vivo* effects of azeliragon on infection control and inflammatory responses, a non-lethal pneumococcal sepsis model was established. Bacterial burdens in blood and lung tissues and serum inflammatory cytokines were assessed after treatment. As shown in Figure 6A, azeliragon (10 mg/kg) significantly reduced bacterial loads (CFU/mL) in the blood compared with the bacterial control group. Vancomycin (10 mg/kg) produced a comparable reduction (Figure 6A). No bacteria were detected in the uninfected control group. In lung tissues, a similar pattern was observed. The bacterial control group showed high pulmonary bacterial burdens, whereas azeliragon treatment markedly decreased CFU/g, indicating effective reduction of lung pathogen load (Figure 6B). Vancomycin treatment also significantly reduced lung bacterial counts (Figure 6B). Both treatment groups differed significantly from the bacterial control group. No bacteria were detected in lungs from the uninfected control group (Figure 6B). Serum inflammatory cytokines were further analyzed. Compared with the bacterial control group, azeliragon (10 mg/kg) significantly decreased serum levels of the pro-inflammatory cytokines IL-6 and TNF-α (Figures 6C,D). IL-6 levels were markedly reduced after azeliragon treatment and were comparable to those in the vancomycin-treated group (Figure 6C). TNF-α showed a similar trend, with azeliragon significantly attenuating infection-induced elevation (Figure 6D). Cytokine levels remained low in the uninfected control group. Collectively, these results demonstrated that, in a non-lethal pneumococcal infection model, azeliragon effectively reduced bacterial burdens in blood and lung tissues and significantly alleviated systemic inflammatory responses. These findings suggested that azeliragon exerted dual *in vivo* effects by controlling pathogen load and modulating inflammation.

## Discussion

The continuous rise in antimicrobial resistance has created substantial barriers to the development of conventional antibiotics that target cell wall synthesis, protein translation, or nucleic acid metabolism (43). This challenge is especially pronounced in highly prevalent Gram-positive pathogens such as *S. pneumoniae*, which readily acquire resistant phenotypes (44). As a result, there is an urgent need to identify small-molecule agents with novel mechanisms of action, non-classical antibacterial targets, or pleiotropic biological effects. In this study, we systematically evaluated the *in vitro* and *in vivo* anti-infective potential of the clinical-stage RAGE inhibitor azeliragon in pneumococcal infection. Azeliragon exhibited stable inhibitory and bactericidal activity against *S. pneumoniae in vitro*. It also significantly improved survival in mouse models of *S. pneumoniae* and concurrently reduced tissue bacterial burdens and inflammatory responses. Together, these findings support azeliragon as a potential non-traditional antibacterial small molecule for the treatment of invasive pneumococcal infections.

At the *in vitro* level, azeliragon demonstrated consistent and reproducible antibacterial activity against *S. pneumoniae*. Its MIC₅₀ and MIC₉₀ values were tightly distributed between 4 and 8 μg/mL, with limited variability among clinical isolates. In contrast, conventional antibiotics such as penicillin and tetracycline showed greater MIC dispersion across the same set of isolates, which was consistent with previous epidemiological studies highlighting resistance heterogeneity in *S. pneumoniae* (14, 45). Prior work has shown that pneumococci acquire resistance through multiple mechanisms, including structural alterations in penicillin-binding proteins and horizontal acquisition of resistance determinants (46). These processes often lead to marked strain-dependent differences in susceptibility to classical antibiotics (47). The relatively narrow MIC distribution observed for azeliragon suggested that its activity may not rely on these traditional resistance pathways, providing preliminary support for its potential utility in resistant settings. Growth kinetic analyses further showed that azeliragon inhibited pneumococcal growth in a clear concentration- and time-dependent manner. At concentrations at or above the MIC, bacterial growth was markedly delayed or completely suppressed. At higher concentrations, the effect shifted from growth inhibition to bactericidal activity. This transition was consistent with the sustained reduction in CFU observed in time-kill assays. Notably, at equivalent multiples of the MIC, the killing kinetics of azeliragon were comparable to those of vancomycin, a bactericidal antibiotic widely used for the treatment of resistant Gram-positive infections (48). These results indicated that azeliragon was not merely bacteriostatic but can exerted sustained bactericidal effects against *S. pneumoniae* at appropriate concentrations.

In complex infection settings, *S. pneumoniae* often persists in a biofilm-associated state. This lifestyle enhances resistance to host immune clearance and markedly reduces antibiotic efficacy (49, 50). Previous studies have shown that pneumococci within biofilms display reduced susceptibility to multiple antimicrobial agents and contribute to chronic infection and treatment failure (50, 51). In this study, azeliragon significantly disrupted the biomass of mature pneumococcal biofilms, with an effect comparable to that of vancomycin. This finding suggested that azeliragon interfered with biofilm stability, thereby increasing its potential utility in clinically relevant infection environments. At the mechanistic level, our results indicated that the bactericidal activity of azeliragon was closely associated with disruption of membrane integrity and induction of oxidative stress. PI staining showed a marked increase in membrane permeability following azeliragon treatment, indicating impairment of the bacterial membrane barrier. In parallel, intracellular ROS levels were significantly elevated. Excessive ROS accumulation is known to cause lipid peroxidation, protein oxidation, and DNA damage and represents a common downstream pathway of several bactericidal mechanisms (52, 53). The concurrent occurrence of membrane damage and oxidative stress observed in this study suggested that these effects acted synergistically to promote bacterial death. This mode of action shares features with several recently reported non-classical antibacterial small molecules [montelukast (36), carmofur (38) and moricin (54)]. However, azeliragon was not a canonical membrane-active agent, and its precise molecular targets remain to be elucidated.

The *in vivo* findings further reinforce the anti-infective potential of azeliragon. In respiratory infection model, azeliragon treatment significantly improved the survival of infected mice, indicating that the compound retains *in vivo* efficacy in a disease context that more closely mimics the natural route of pneumococcal infection. Meanwhile, in mouse sepsis models induced by *S. pneumoniae* strains of different origins, azeliragon significantly improved survival in a dose-dependent manner. Mortality from invasive pneumococcal infection is driven not only by pathogen burden but also by excessive inflammatory responses and subsequent organ damage (55, 56). Therefore, strategies based solely on bacterial clearance often fail to achieve optimal outcomes in severe disease. In a non-lethal infection model, azeliragon not only reduced bacterial loads in blood and lung tissues but also significantly decreased serum levels of the key pro-inflammatory cytokines IL-6 and TNF-α. These findings were consistent with previous reports describing RAGE as an amplifier of infection-induced inflammatory responses (57, 58). They suggested that azeliragon may improve host outcomes *in vivo* through a dual mechanism that combines direct antibacterial activity with suppression of inflammatory cascades. In summary, this study provided the first systematic evidence that the RAGE inhibitor azeliragon exerted direct antibacterial activity against *S. pneumoniae* and confers *in vivo* protection in models of pneumococcal sepsis. Compared with conventional antibiotics, azeliragon may act through mechanisms distinct from classical targets while simultaneously modulating host inflammatory responses. This dual activity highlights its potential as a novel therapeutic strategy for invasive pneumococcal infections. Although the precise molecular targets and its potential synergy with existing antibiotics require further investigation, the present findings establish a solid foundation for future mechanistic and translational studies.

## Conclusion

This study systematically demonstrated that azeliragon exhibited stable *in vitro* inhibitory and bactericidal activity against *S. pneumoniae*, based on analyses of antimicrobial activity, killing kinetics, mechanisms of action, and *in vivo* efficacy. Azeliragon effectively reduced pneumococcal biofilms and exerted bactericidal effects through disruption of membrane integrity and induction of oxidative stress. In animal infection models, azeliragon significantly improved host survival, reduced tissue bacterial burdens, and attenuated inflammatory responses. As a non-classical antibacterial small molecule, azeliragon may act through mechanisms distinct from those of conventional antibiotics and offered combined potential for pathogen control and host protection. These findings provide a foundation for further mechanistic studies and translational development of azeliragon for invasive pneumococcal infections and offer new insights into the discovery of novel antibacterial agents.

## Data Availability

Publicly available datasets were analyzed in this study. This data will be made available on request.
